# Tsp2 Facilitates Tumor-associated Fibroblasts Formation and Promotes Tumor Progression in Retroperitoneal Liposarcoma

**DOI:** 10.7150/ijbs.70083

**Published:** 2022-08-01

**Authors:** Chang Xu, Liang Yan, Xiaoya Guan, Zhen Wang, Jianhui Wu, Ang Lv, Daoning Liu, Faqiang Liu, Bin Dong, Min Zhao, Ling Jia, Xiuyun Tian, Chunyi Hao

**Affiliations:** 1 Key Laboratory of Carcinogenesis and Translational Research (Ministry of Education), Department of Hepato-Pancreato-Biliary Surgery, Peking University Cancer Hospital & Institute, Beijing, China.; 2 Key Laboratory of Carcinogenesis and Translational Research (Ministry of Education), Central Laboratory, Peking University Cancer Hospital & Institute, Beijing, China.; 3 Key Laboratory of Carcinogenesis and Translational Research (Ministry of Education), Department of Pathology, Peking University Cancer Hospital & Institute, Beijing, China.

**Keywords:** retroperitoneal liposarcoma, Tsp2, tumor-associated fibroblasts, tumor microenvironment, tumor progression

## Abstract

Retroperitoneal liposarcoma (RLPS) is the most common subtype of retroperitoneal soft tissue sarcoma, characterized by a high recurrence rate and insensitivity to radiotherapy and chemotherapy. The function of tumor microenvironmental components, especially tumor-associated fibroblasts (TAFs), remains unclear in RLPS. The crosstalk between tumor cells and stromal cells should be clarified for therapy target discovery in RLPS. In this study, we demonstrated that TAFs from dedifferentiated liposarcoma (DDLPS) could attract LPS cells and promote their proliferation and migration. However, although α-SMA is positively expressed in RLPS, its expression does not indicate prognosis. By screening differentially expressed genes, performing Oncomine visualization, TCGA gene expression correlation analysis and qPCR verification, we determined that thrombospondin-2 (THBS2) gene expression was related to TAFs. The expression of Tsp2 protein, which was encoded by THBS2, was correlated with α-SMA expression, and it was an independent predictive factor for disease-free survival and recurrence-free survival in patients with RLPS. *In vitro*, Tsp2 facilitated the transformation of bone marrow-derived fibroblasts (BMFs) to TAFs and promoted the malignant biological behaviors of LPS cells by activating the MAPK/MEK/ERK pathway. Therefore, suppression of Tsp2 is expected to be a promising treatment method for RLPS patients.

## Introduction

Retroperitoneal soft tissue sarcomas (RSTSs) are a group of rare mesenchymal malignancies 1. Retroperitoneal liposarcoma (RLPS) is the most common subtype in RSTSs, and it is classified by morphological and genetic characteristics into well-differentiated liposarcoma (WDLPS), dedifferentiated liposarcoma (DDLPS), myxoid liposarcoma (MLPS), and pleomorphic liposarcoma (PLS) 2. It is difficult to achieve complete resection in RLPS due to its location and characteristics of local infiltration, and local recurrence is common 3. Incomplete resection and recurrence are correlated with poor prognosis 4. Additionally, it remains controversial whether RLPS patients benefit from systemic chemotherapy and radiotherapy 5,6. Therefore, it is necessary to explore new therapeutic targets from tumor cells or the tumor microenvironment (TME) so as to improve RLPS treatment.

Tumor-associated fibroblasts (TAFs) are the most common cell type in the TME and are important for tumorigenesis and progression 7,8. TAFs can secrete cytokines such as TGF-β, IL-6, CXCL12 9 and interact with other cells, thereby promoting malignant biological behavior of cancer cells, and the formation of immunosuppressive microenvironment 10-12. On the other hand, TAFs secrete collagen, fibronectin (FN), matrix metalloproteinases (MMPs) and other substances to remodel extracellular matrix (ECM), and facilitate tumor progression 13,14. TAFs are often considered as a type of activated normal fibroblasts, which are induced by TGF-β, PDGF, CCL2, and other cytokines secreted by tumor cells 15-17. Activated TAFs have a typical spindle shape and can express markers such as α-smooth muscle actin (α-SMA) and fibroblast-activation protein (FAP) 17-19. Resting fibroblasts are multi-derived and can be converted from bone marrow mesenchymal stem cells, adipose stem cells, epithelial and endothelial cells 11,19,20. Therefore, it may be more effective to reduce TAFs formation and their cancer-promoting effects through inhibiting TAF-inducing factors than directly targeting TAFs.

Harati et al. found that TAFs isolated from MLPS and PLS promoted cell proliferation and doxorubicin resistance in SW872 cells 21. However, the effect of TAFs and the crosstalk between TAFs and tumor cells have not yet been clarified in RLPS. In this study, we aimed to reveal the role of TAFs in RLPS and to investigate the mechanisms by which Tsp2 promotes TAFs formation and tumor progression. Our findings are helpful to profile the TME in RLPS and demonstrate Tsp2 as a potential prognostic factor and therapeutic target.

## Materials and Methods

### Patients and samples

Overall, 112 RLPS and four adipose samples from patients who underwent surgery at Peking University Cancer Hospital were included in immunohistochemical (IHC) staining experiments. These patients were pathologically diagnosed as RLPS by performing HE staining and observing the histological morphology. WDLPS resembles mature adipose tissue, but typically shows fibrous septa, nuclear atypia and nuclear enlargement. DDLPS typically presents as undifferentiated pleomorphic or spindle cells, and is usually non-adipose-derived sarcoma. The detailed clinicopathological information is shown in Table S1. Additionally, 40 DDLPS and 10 adipose tissues were used in quantitative real-time PCR (qPCR) to evaluate mRNA expression. This study was approved by the Institutional Review Board of Peking University Cancer Hospital (2019KT19). Written informed consent was obtained from each patient.

### Isolation and characterization of TAFs

TAFs were isolated from freshly resected WD/DDLPS tissues. All tissues were cut into 1-3-mm^3^ blocks. After incubated with 1640 medium at 37 °C for 1 day, sufficient medium was added. When a large number of cells can be observed, the tissues were removed, and the spindle cells were separated owing to the principle that fibroblasts are more sensitive to trypsin. Purified spindle cells were cultured in DMEM/F12 with 10% FBS. Immunofluorescence and qPCR were used to assess the expression of FAP and α-SMA.

### Immunofluorescence

Liposarcoma (LPS) cells and TAFs were seeded in confocal chambers and fixed with paraformaldehyde for 20 min. The cells were blocked with 1% BSA for 1h, followed by incubation with anti-FAP antibody (AF5344, 1:200, Affinity), anti-α-SMA antibody (ab7817, 1 μg/ml, Abcam), or anti-MDM2 antibody (ab38618, 1:200, Abcam) overnight. Secondary antibodies were incubated with cells for 1 h and nucleus was stained with DAPI.

### Quantitative real-time PCR

Total RNA was extracted from cells and tissues using TRIzol (Thermo Fisher Scientific, USA). After reverse transcription, qPCR was performed to detect the relative mRNA expression of FAP, ACTA2, THBS2, and other genes in the samples. The primers are listed in Table S2.

### Cells culture and co-culture

LPS cell lines were purchased from ATCC (Manassas, VA, USA). Bone marrow-derived fibroblasts (BMFs) were isolated from bone marrow stromal cells in adult volunteers without systemic disease by the method reported previously 22. LPS cells and BMFs were cultured in RPMI 1640 with 10% FBS. TAFs were cultured in DMEM/F12 with 10% FBS. ①TAF and BMF-conditioned media (CM) and co-culture with LPS cells: TAFs and BMFs were plated to reach confluence before being cultured in DMEM/F12 for 24h, and the media was collected and filtered with sterile membrane (Millipore, PR05538). TAF-CM and BMF-CM were used to cultivate LPS cells for 24h, and then LPS cells were collected to evaluate biological behaviors. ②LPS-CM and co-culture with fibroblasts: LPS cells were plated to reach confluence before being cultured in RPMI 1640 for 24h, and the media was filtered through sterile membrane. LPS-CM was used to cultivate BMFs for 24h, and then fibroblasts were harvested to detect the expression of TAF-related genes.

### Cell proliferation assay

LPS cells treated under different conditions were seeded in 96-well plates. Then, 10 μl CCK8 reagent (Dojindo, Japan) was added into each well at 0, 24, 48, 72, and 96 h. After 2-3 h of incubation, a microplate reader was used to measure absorbance at 450 nm.

### Cell migration assay

LPS cells treated under different conditions were mixed in RPMI 1640 and seeded into the upper chamber (3422, Corning), and RPMI 1640 with 20% FBS was added to the lower chamber. After 24 h of migration at 37 °C, cells on the membrane of the upper chamber were fixed with 4% paraformaldehyde and stained with 0.1% crystal violet. The stained cells were photographed in five fields, and the number of migrating cells was compared.

### Cell chemotaxis assay

LPS cells were seeded into the upper chamber (3422, Corning) with DMEM/F12 containing 2% FBS, and BMFs or TAFs were added into the lower chamber with DMEM/F12 containing 20% FBS. DMEM/F12 with 20% FBS was added to the lower chamber as control. The ratio of the number of LPS and BMFs/TAFs was 1:1. After 24 h of chemotaxis at 37 °C, the cells on the membrane were stained and the number was compared.

### IHC staining and evaluation

The sections of tumor tissues or adipose tissues were deparaffinized in xylene, hydrated in gradient alcohol, and incubated in 3% hydrogen peroxide. Antigen retrieval was conducted under high pressure or microwave, followed by goat serum to block the slices. Slices were incubated with α-SMA antibody (ab7817, 0.05 μg/ml, Abcam), Tsp2 antibody (PA5-80123, 2 μg/ml, Invitrogen), and Ki67 antibody (ab15580, 1:100, Abcam) overnight and then incubated with secondary antibody. DAB was used to visualize the slices, and hematoxylin was used to stain the nuclei.

All slices were examined and scored by two independent pathologists. Expression of α-SMA was defined as positive when the staining percentage was >10%. A small number of α-SMA positive smooth muscle cells in the vessel wall that can form tubular structures were not considered as positive results. The expression of Tsp2 was evaluated by the immunoreactivity score (IRS) according to the percentage of positive cells (PP) and staining intensity (SI). PP was scored as 0 (negative), 1 (<25%), 2 (25%-75%), 3 (>75%). SI was scored as 0 (negative), 1 (weak), 2 (moderate), 3 (strong). IRS=0/1 was defined as negative expression, and IRS>1 as positive expression.

### Differentially expressed genes screening, functional enrichment, expression correlation analysis and qPCR verification

The GSE21122 dataset was downloaded from the Gene Expression Omnibus database (https://www.ncbi.nlm.nih.gov/geo/). This dataset contained high-throughput gene expression data from 89 LPS specimens (46 DDLPS, 20 MLPS, 23 PLS) and nine adipose samples. The GEO2R module was used to screen the differentially expressed genes (DEGs) between DDLPS and adipose tissue. The screening criteria were FDR<0.05, and |log_2_ fold change|>2. DAVID online software (https://david.ncifcrf.gov/) was used to perform functional enrichment analysis based on Gene Ontology (GO) and the Kyoto Encyclopedia of Genes and Genomes (KEGG) database.

The Search Tool for the Retrieval of Interacting Genes/Proteins (STRING, https://www.string-db.org/) was used to analyze the interaction between proteins and construct protein-protein interaction (PPI) network. The MCODE plugin of Cytoscape was used to identify the gene modules. Oncomine (https://www.oncomine.or) was used to visualize the expression of key genes. Correlation analysis between gene expression in the Cancer Genome Atlas (TCGA) was performed using cBioPortal for Cancer Genomics (http://www.cbioportal.org/). Ten adipose and 40 DDLPS tissues were tested by qPCR to clarify DEGs expression and verify the expression correlation.

### Cell transfection

Three lentiviral shRNA vectors against Tsp2 and control lentivirus were transfected into SW872 cells to knockdown Tsp2 expression. 93T449 and 94T778 cells were infected with Tsp2 overexpression lentivirus and a control lentivirus. All lentiviruses were obtained from Shanghai Gene Chem Co., Ltd. The efficiency of knockdown or overexpression was verified by qPCR and western blotting.

### Western blotting

Total protein was extracted from cells, and the concentration was determined using a BCA kit (Thermo Fisher Scientific, USA). Proteins were separated by SDS-PAGE and transferred onto PVDF membranes. Membranes were blocked with Tris-buffered saline-Tween solution containing 5% skim milk. Next, the PVDF membranes were incubated with primary antibodies (Table S3) overnight and secondary antibodies for 1h. Finally, a chemiluminescence detection system was used to detect protein bands.

### ELISA

A double-antibody sandwich ELISA kit (cat.TAE-890h, Anoric Bio-tech, China) was used. According to the manufacturer's instructions, Tsp2 protein levels in conditioned medium of LPS cells with Tsp2 overexpression or Tsp2 knockdown was assayed by ELISA. After the antibody-antigen complex was formed, the substrate was added for the color reaction. Finally, stopping solution was added, and the staining product was measured by a microplate reader at 450 nm.

### Cell apoptosis assay

According to the manufacturer's instructions, 5 μL each of Annexin V and PI solution (Annexin V, 633 Apoptosis Detection Kit, Dojindo, Japan) was added to the cell suspension prepared with 1×Annexin V binding buffer. Two single positive controls and a negative control were also prepared to adjust for compensation. After 15 min of staining, flow cytometry was used to measure the proportion of apoptotic cells.

### Xenograft model

SW872 shCtrl and SW872 shTsp2 cells were resuspended to 1×10^8^ cells/ml and injected subcutaneously into the back of NOD/SCID mice (each injection was 100 μL). Each group included 5 mice. The tumor volume was calculated according to the formula V=1/2 length × width^2^ twice a week, and the tumor growth curve was drawn. After 60 days, the mice were sacrificed, and tumors were removed and photographed. Tumor tissues were tested by qPCR or IHC to analyze the expression of molecules. All studies were conducted in compliance with the animal ethics and welfare requirements (ethics approval number: EAEC2018-06).

### Protein microarray analysis

According to the manufacturer's instructions, the Proteome Profiler^TM^ Array-Human Phospho-Kinase Array Kit (ARY003B, R&D Systems) was used to assess the changes in key targets in tumor-related signaling pathways after Tsp2 overexpression.

### Statistical analysis

Statistical analysis were performed using SPSS 17.0 and GraphPad Prism 8. Continuous variables are represented as mean±SD, and categorical variables are represented as numbers and percentages. Student's t-test, Mann-Whitney U test, and ANOVA test were used to compare continuous variables, and chi-square test was used to compare categorical variables. Spearman method was used to analyze the correlation between continuous variables that do not conform to the normal distribution.

Eight patients lost to follow-up and 4 patients died of intraoperative, perioperative or postoperative complications, so 100 patients were included for survival analysis. Kaplan-Meier survival curves were analyzed using the log-rank test. Univariate and multivariate analysis were performed using the Cox proportional hazard regression model to identify independent prognostic factors affecting overall survival (OS), disease-free survival (DFS), and recurrence-free survival (RFS). Differences were considered significant at p<0.05.

## Results

### Isolation and characterization of TAFs from RLPS

**Figure 1:**
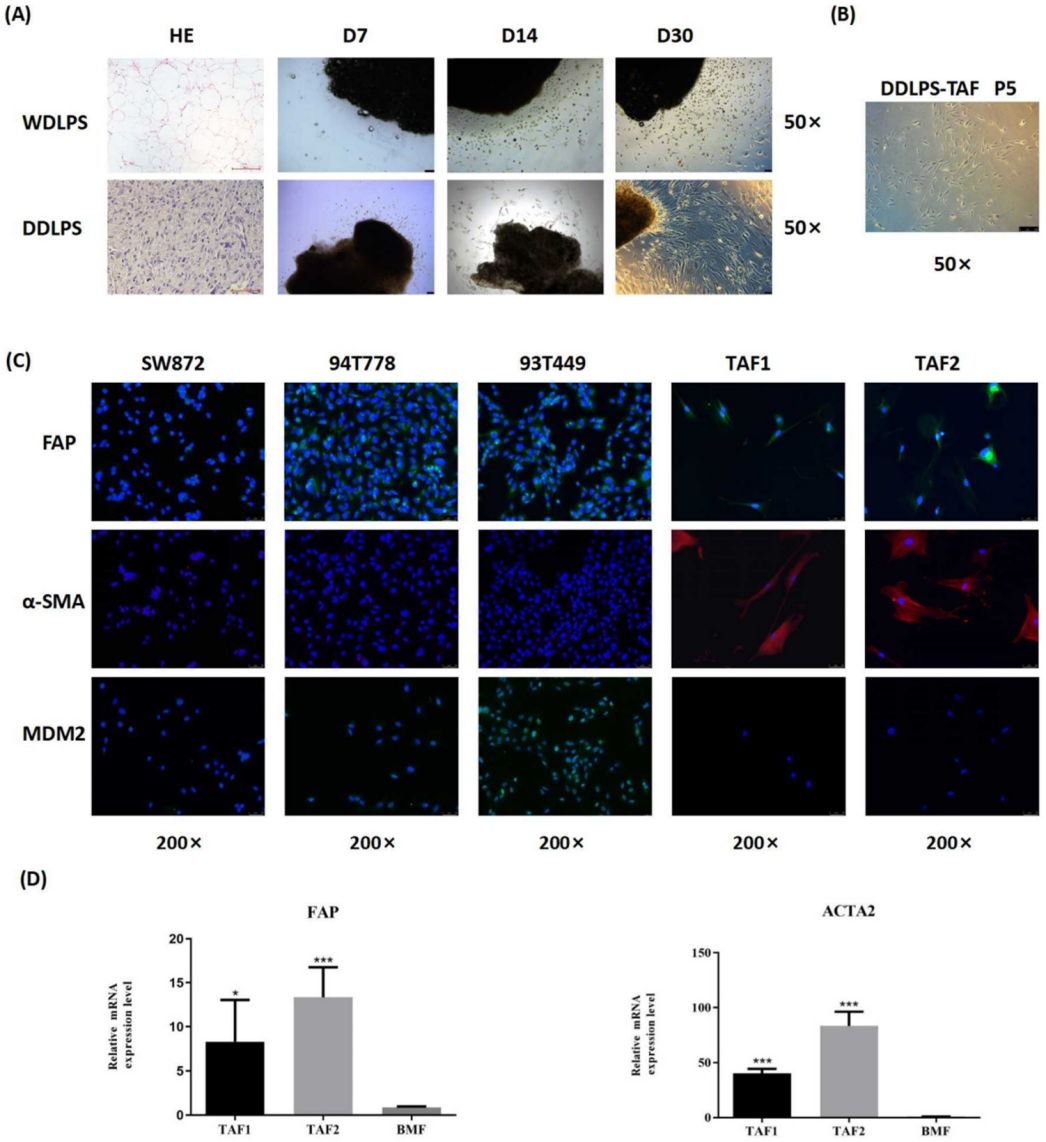
** Isolation and characterization of TAFs from RLPS. (A-B)** Morphology and growth of cells migrated from WDLPS and DDLPS. Magnifications: ×50. **(C)** Immunofluorescence staining of LPS cells and TAFs with FAP (green), α-SMA (red), MDM2 (green) and counterstained with DAPI (blue). Magnifications: ×200. **(D)** qPCR analysis of FAP and ACTA2 expression in BMFs and TAFs. Statistical tests: Student's t tests.

Two WDLPS and 2 DDLPS tissues were used to isolate TAFs. The clinicopathological characteristics of the patients and the culture results of TAFs are shown in Table S4. After 30 days of culture, spindle cells were observed around the DDLPS tissues, but most cells that migrated from the WDLPS tissues were round (Fig. 1A). Spindle cells isolated from DDLPS can be purified and stably cultured to the fifth generation (Fig. 1B). No stable spindle cells were purified from cells that migrated from the WDLPS tissues. Spindle cells isolated from 2 DDLPS tissues were named as TAF1 and TAF2.

Immunofluorescence staining showed that LPS cells (93T449, 94T778, and SW872) did not express α-SMA, and they expressed FAP weakly, while TAFs were FAP- and α-SMA-positive (Fig. 1C). MDM2 expression was observed in 93T449 and 94T778 cells, whereas TAFs had no MDM2 protein expression, suggesting that the purified TAFs did not contain RLPS tumor cells. Quantitative real-time PCR analysis showed that compared with BMFs, the relative mRNA levels of FAP and ACTA2 in TAFs increased significantly (Fig. 1D). Therefore, we successfully isolated and identified primary TAFs from RLPS. TAF1 was used in the subsequent experiments.

### TAFs from DDLPS promoted proliferation, migration and chemotaxis of three LPS cell lines

**Figure 2:**
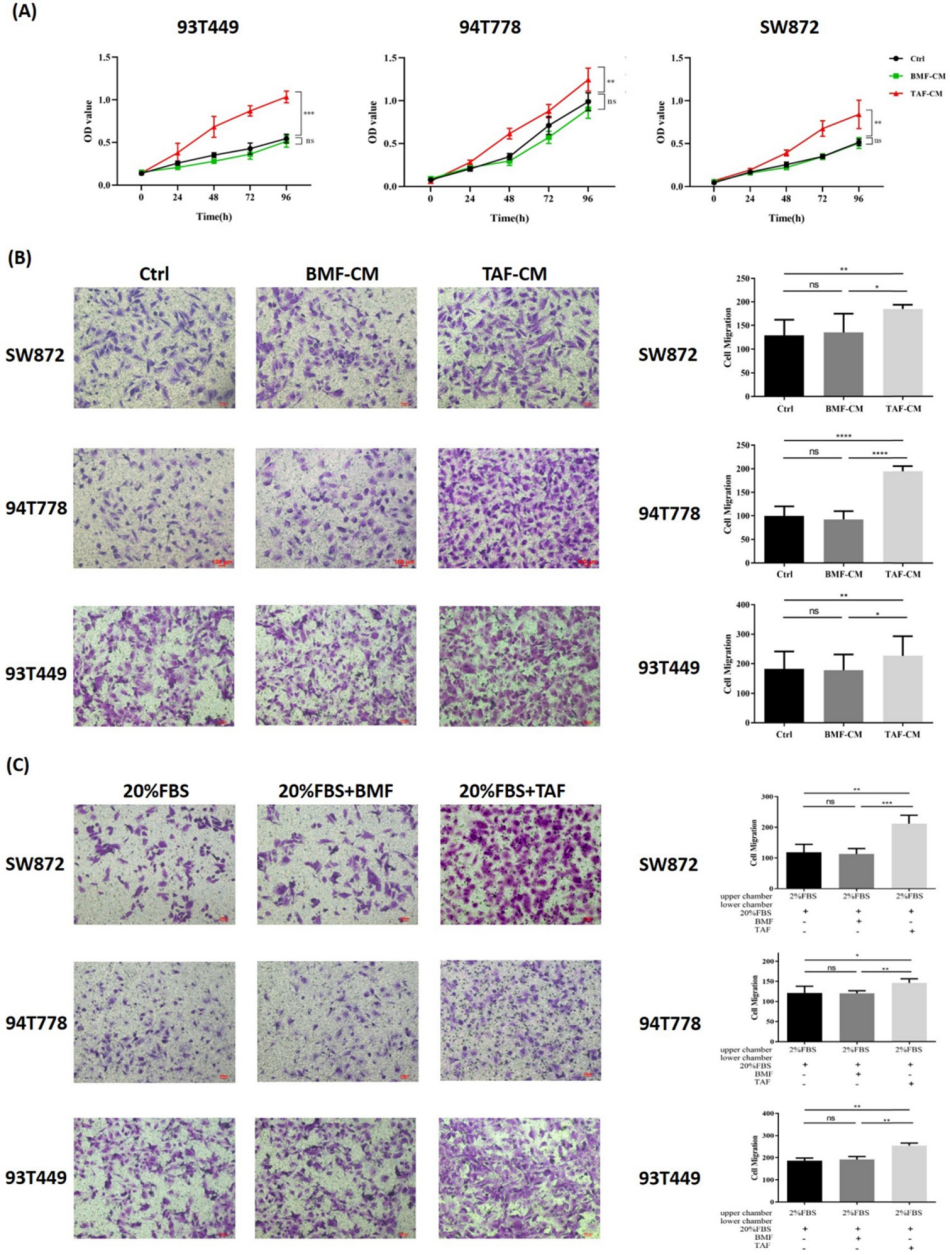
** TAFs from DDLPS promoted proliferation, migration and chemotaxis of LPS cells. (A-B)** LPS cells cultured in TAF-CM had a higher proliferation rate and stronger migration ability than cultured in DMEM/F12 or BMF-CM (p<0.05). **(C)** Compared with the controls and BMFs, TAFs significantly promoted the chemotaxis of LPS cells (p<0.05). Statistical tests: Student's t tests.

Co-culture experiments were conducted to investigate the effect of TAFs on the biological behaviors of 93T449, 94T778, and SW872 cells. After 24h of co-culture, three LPS cell lines cultured in TAF-CM had a higher proliferation rate (Fig. 2A, p<0.05) and stronger migration ability (Fig. 2B, p<0.05) than LPS cells cultured in DMEM/F12 or BMF-CM. Compared with the control and BMFs, TAFs significantly promoted the chemotaxis of the three LPS cell lines (Fig. 2C, p<0.05). These results indicated that TAFs derived from DDLPS could promote the proliferation and migration of LPS cells and could attract LPS cells through chemotaxis.

### TAF biomarker expression in RLPS and its clinical relevance

**Figure 3:**
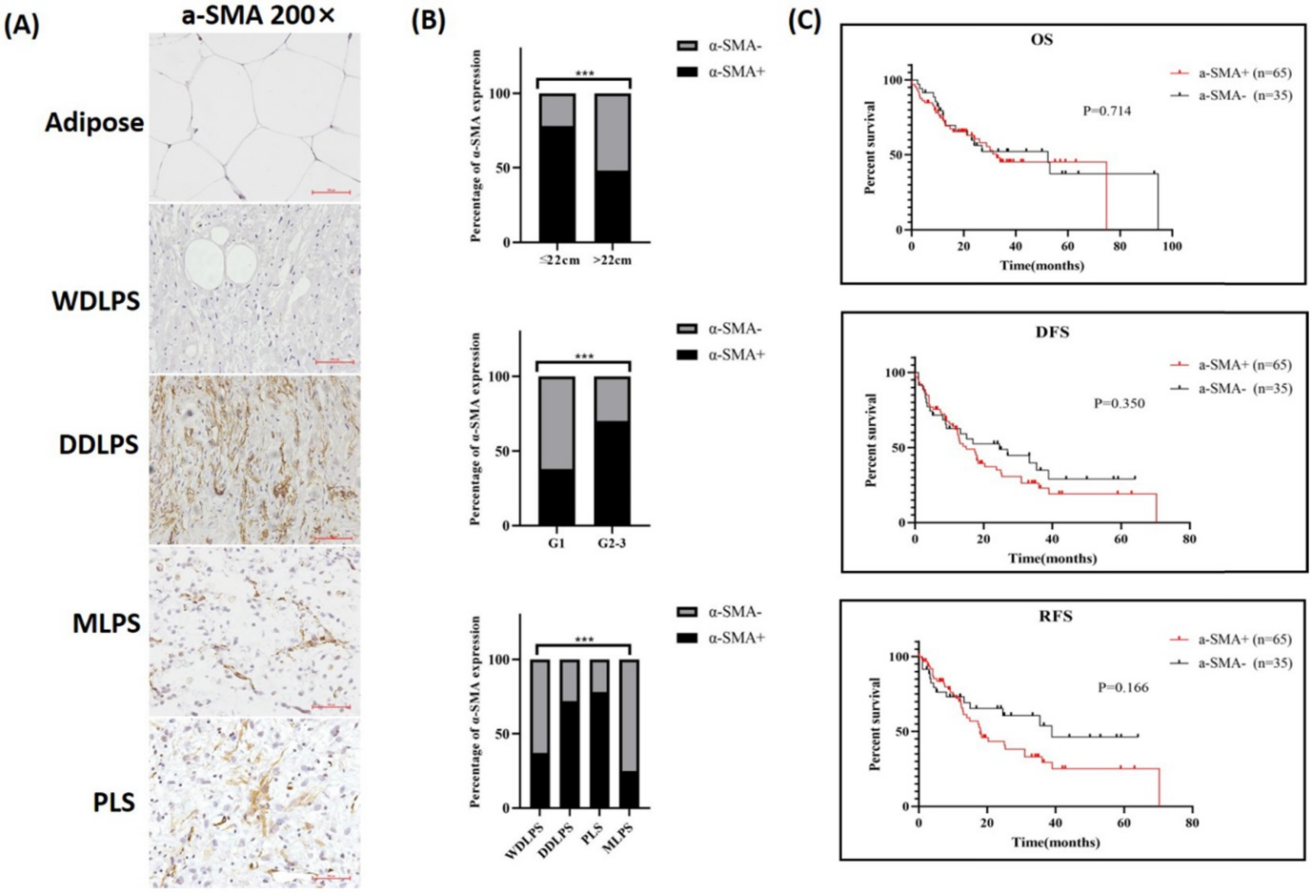
** α-SMA expression and clinical relevance in RLPS. (A)** Typical expression of α-SMA in adipose and RLPS tissues. **(B)** Correlations between α-SMA expression and clinicopathological features in RLPS patients. **(C)** α-SMA positive expression had no correlation with OS, DFS and RFS of RLPS. Statistical tests: Chi-square test and Kaplan-Meier survival analysis.

Since FAP expression was observed in some LPS cells, α-SMA was chosen as a suitable marker for TAFs and stained in RLPS and adipose tissues. The typical expression of α-SMA in adipose and different subtypes of RLPS are shown in Fig. 3A. Of the 112 tumor specimens, 71 were α-SMA-positive (63.4%), while four adipose tissues were α-SMA negative. As shown in Fig. 3B and Table 1, positive α-SMA expression was related to tumor diameter (p=0.002), grade (p=0.004), and subtype (p=0.005).

One hundred RLPS patients received postoperative follow-up and were divided into two groups based on α-SMA expression for survival analysis (Fig. 3C). The median OS time was 32.6 months and 52.3 months in patients with positive α-SMA or negative α-SMA expression, respectively. The median DFS and RFS time in the positive α-SMA group was 14.9 months and 18.0 months, and was 24.6 months and 38.9 months in the negative α-SMA expression group. The OS, DFS, and RFS in the α-SMA positive group were poorer, though the differences were not statistically significant (p=0.714, p=0.350, p=0.166, respectively). Therefore, α-SMA was highly expressed in RLPS, but it did not effectively suggest the prognosis.

### Screening and validation of DEGs related to TAFs

**Figure 4:**
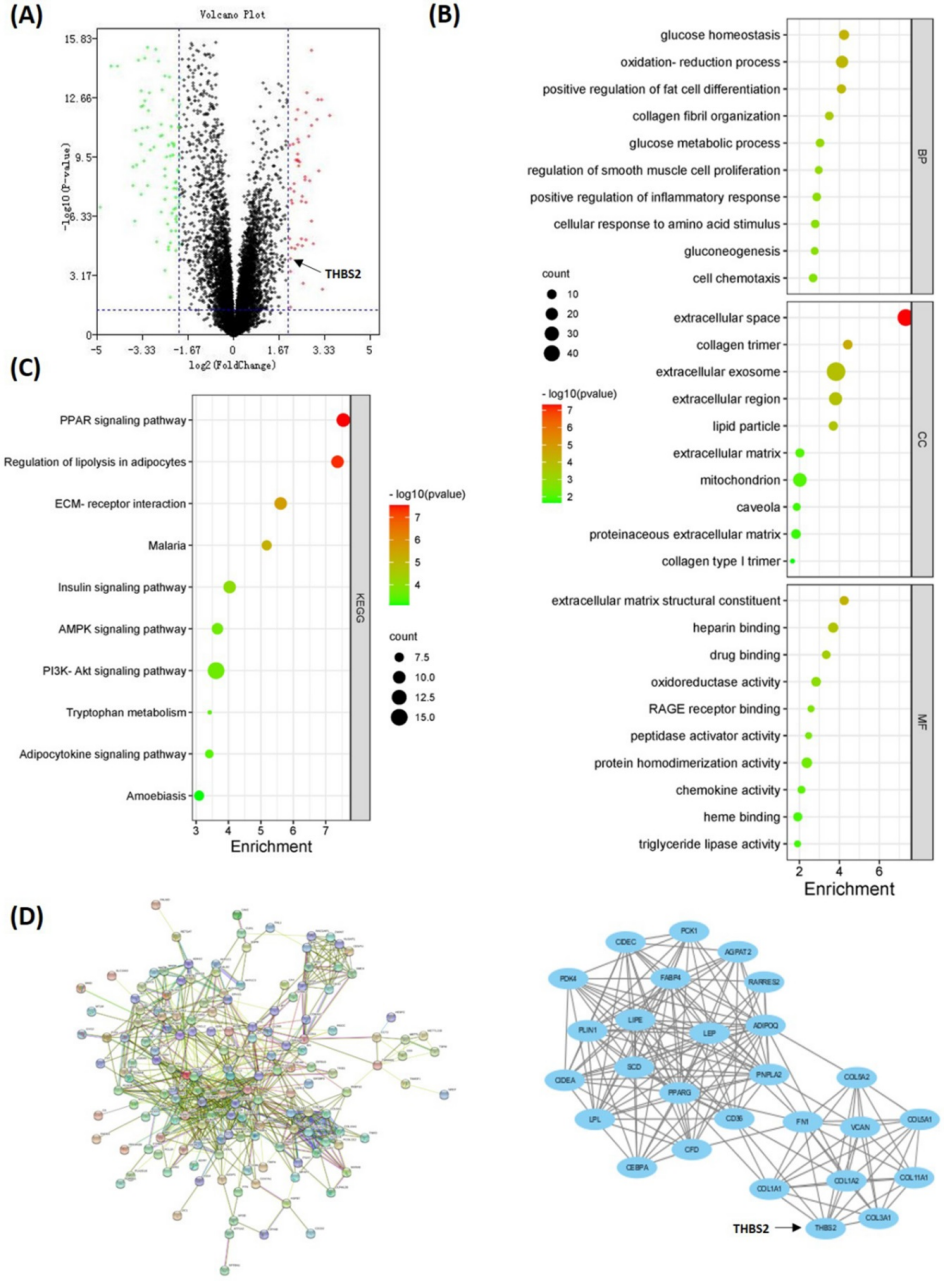
** DEGs between DDLPS and adipose samples. (A)** Volcano map of DEGs between DDLPS and adipose tissues. **(B-C)** GO and KEGG analysis of DEGs by DAVID online software (https://david.ncifcrf.gov/). **(D)** PPI network and the key cluster in DEGs. Implemented by STRING website (https://www.string-db.org/) and Cytoscape software.

To investigate the DEGs related to TAFs, we compared the gene expression profile data of 46 DDLPS and 9 adipose samples in the GSE21122 dataset. Overall, 193 DEGs were identified, wherein 47 were upregulated and 146 were downregulated in DDLPS (Fig. 4A). GO analysis showed that DEGs were enriched in categories such as collagen fibril organization, smooth muscle cell proliferation regulation, extracellular matrix structural constituent, extracellular space, and collagen trimer (Fig. 4B). KEGG analysis showed that DEGs were enriched in pathways such as PPAR, regulation of lipolysis in adipocytes, and ECM-receptor interaction (Fig. 4C). All DEGs were used to construct a PPI network. And the key cluster, including 27 nodes, was calculated using Cytoscape (Fig. 4D).

**Figure 5:**
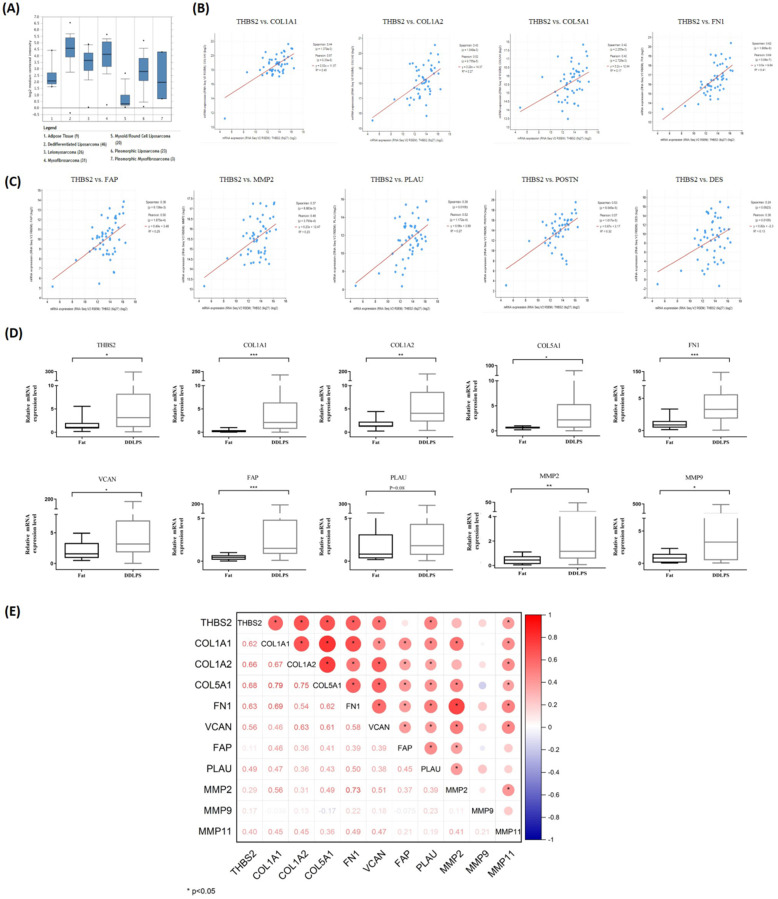
** Screening and validation of DEGs related to TAFs. (A)** Visualization of THBS2 expression in LPS by Oncomine database (http://www.oncomine.org/). **(B-C)** THBS2 expression was positively correlated with ECM-related genes and TAFs-related genes (p<0.05). Data sets and analysis can be found on cBioPortal for Cancer Genomics (www.cbioportal.org). **(D)** Compared with adipose, the relative mRNA expression of THBS2, COL1A1, COL1A2, COL5A1, FN1, VCAN, FAP, MMP2 and MMP9 was higher in DDLPS (p<0.05). **(E)** The expression of THBS2 was positively correlated with the expression of ECM-related genes COL1A1, COL1A2, COL5A1, FN1, VCAN, and was positively correlated with the expression of TAFs secretase PLAU and MMP11 (p<0.05). Statistical tests: Mann-Whitney U test and Spearman correlation analysis.

The Oncomine database was used to visualize the expression of 27 key genes (e.g., PPARG, CEBPA, THBS2, COL1A1, FN1, and VCAN) in LPS. Compared with adipose tissue, thrombospondin-2 (THBS2) gene was highly expressed in DDLPS and PLS, and weakly expressed in MLPS, consistent with α-SMA expression in RLPS tissues (Fig. 5A). To clarify whether THBS2 expression was related to TAFs, cBioportal was used to analyze the correlation of gene expression in 50 DDLPS patients in the Adult Soft Tissue Sarcomas (TCGA, Cell2017) dataset. THBS2 expression was positively correlated with the expression of ECM-related genes COL1A1, COL1A2, COL5A1, FN1 (p<0.05, Fig. 5B) and TAF-related genes FAP, MMP2, PLAU, POSTN, and DES (p<0.05, Fig. 5C). Next, we performed qPCR on ten adipose tissues and 40 DDLPS tissues to verify the database results. Compared with adipose tissues, the relative mRNA expression of THBS2, COL1A1, COL1A2, COL5A1, FN1, VCAN, FAP, MMP2, and MMP9 increased in DDLPS tissues (p<0.05, Fig. 5D), but the increase of PLAU (p=0.08) and MMP11 (data not shown) was not significant (Fig. 5D). Spearman correlation analysis revealed that the expression of THBS2 was positively correlated with the expression of ECM-related genes COL1A1, COL1A2, COL5A1, FN1, VCAN, and was positively correlated with the expression of TAFs secretase PLAU and MMP11 (p<0.05, Fig. 5E). These results indicated that THBS2 was correlated with the expression of TAF-related genes and ECM-related genes in DDLPS. THBS2 may be related to the high infiltration of TAFs in DDLPS.

### Tsp2 protein expression in RLPS and its clinical relevance

**Figure 6:**
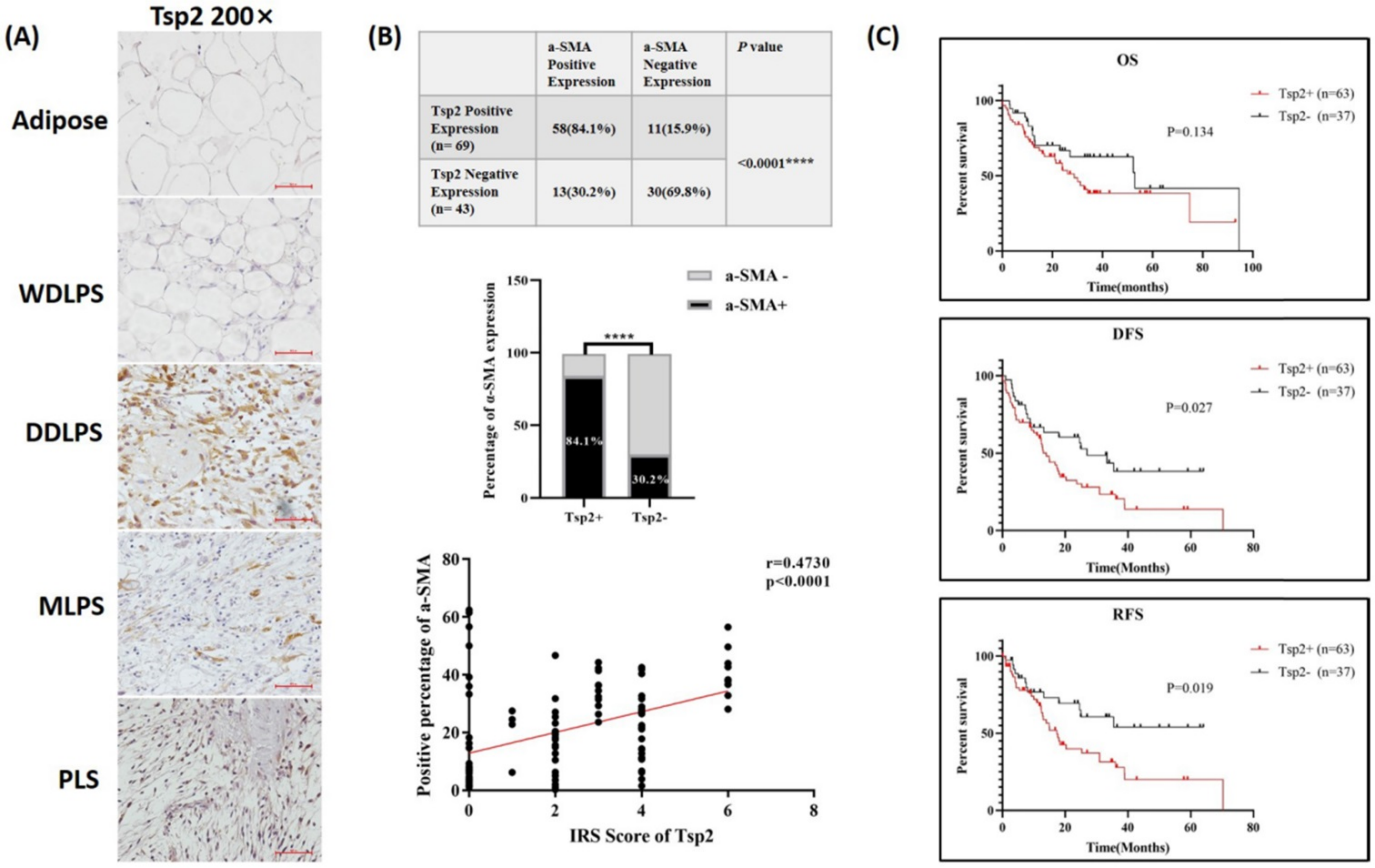
** Tsp2 expression and clinical significance in RLPS. (A)** Typical expression of Tsp2 in adipose and RLPS tissues. **(B)** Tsp2 expression was positively correlated with α-SMA (p<0.0001). **(C)** Positive Tsp2 expression was correlated with poorer DFS and RFS in RLPS (p<0.05). Statistical tests: Chi-square test, Spearman correlation analysis and Kaplan-Meier survival analysis.

Next, Tsp2 protein, which was encoded by THBS2, was stained in RLPS and adipose tissues, and its clinical relevance was assessed. The typical expression of Tsp2 was shown in Fig. 6A. Tsp2 was mainly expressed in liposarcoma cells, and also expressed in a small proportion of TAFs. Tsp2 was positive in 61.6% (69/112) of RLPS patients, while four adipose tissues were Tsp2 negative. As shown in Table S5, Tsp2 expression was higher in patients with tumors diameter ≤22 cm and high-grade RLPS, but the difference was not significant (p=0.052, p=0.073, respectively).

Among patients with positive Tsp2 expression, 84.1% (58/69) were α-SMA-positive, and 15.9% (11/69) were negative. Among patients with negative Tsp2 expression, 30.2% (13/43) were α-SMA positive and 69.8% (30/43) were negative (Fig. 6B). The expression of Tsp2 and α-SMA were significantly related (p<0.0001). The correlation coefficient between the IRS score of Tsp2 and the percentage of α-SMA positive cells was 0.473 (p<0.0001, Fig. 6B). Therefore, Tsp2 expression was positively correlated with α-SMA expression (p<0.0001).

In the survival analysis (Fig. 6C), the median OS time was 28.7 months in Tsp2-positive patients and 53.0 months in negative patients. The median DFS and RFS were 14.0 months and 17.4 months in Tsp2-positive patients and 27.0 months and undefined (accumulate survival rate >50%) in Tsp2-negative patients. Positive Tsp2 expression was markedly correlated with poorer DFS and RFS (p=0.027 and p=0.019, respectively).

Univariate survival analysis showed Tsp2 expression (p=0.030), higher (G2 or G3) grade tumors (p=0.033, p=0.014), DDLPS (p=0.030), MLPS (p=0.018), and organ invasion (p=0.004) were predictive factors for DFS. Cox multivariate survival analysis further revealed that Tsp2 expression (p=0.0497) and organ invasion (p=0.008) were independent predictive factors for DFS (Table 2).

Univariate survival analysis suggested that Tsp2 positive (p=0.022), G3 grade tumors (p=0.022), MLPS (p=0.008), lymph node metastasis (p=0.040), and organ invasion (p=0.006) were predictive factors for RFS. Cox multivariate survival analysis showed that Tsp2 (p=0.033) and lymph node metastasis (p=0.009) were independent predictive factors for RFS (Table 3).

### Tsp2 promoted BMFs transformation to TAFs *in vitro*

**Figure 7:**
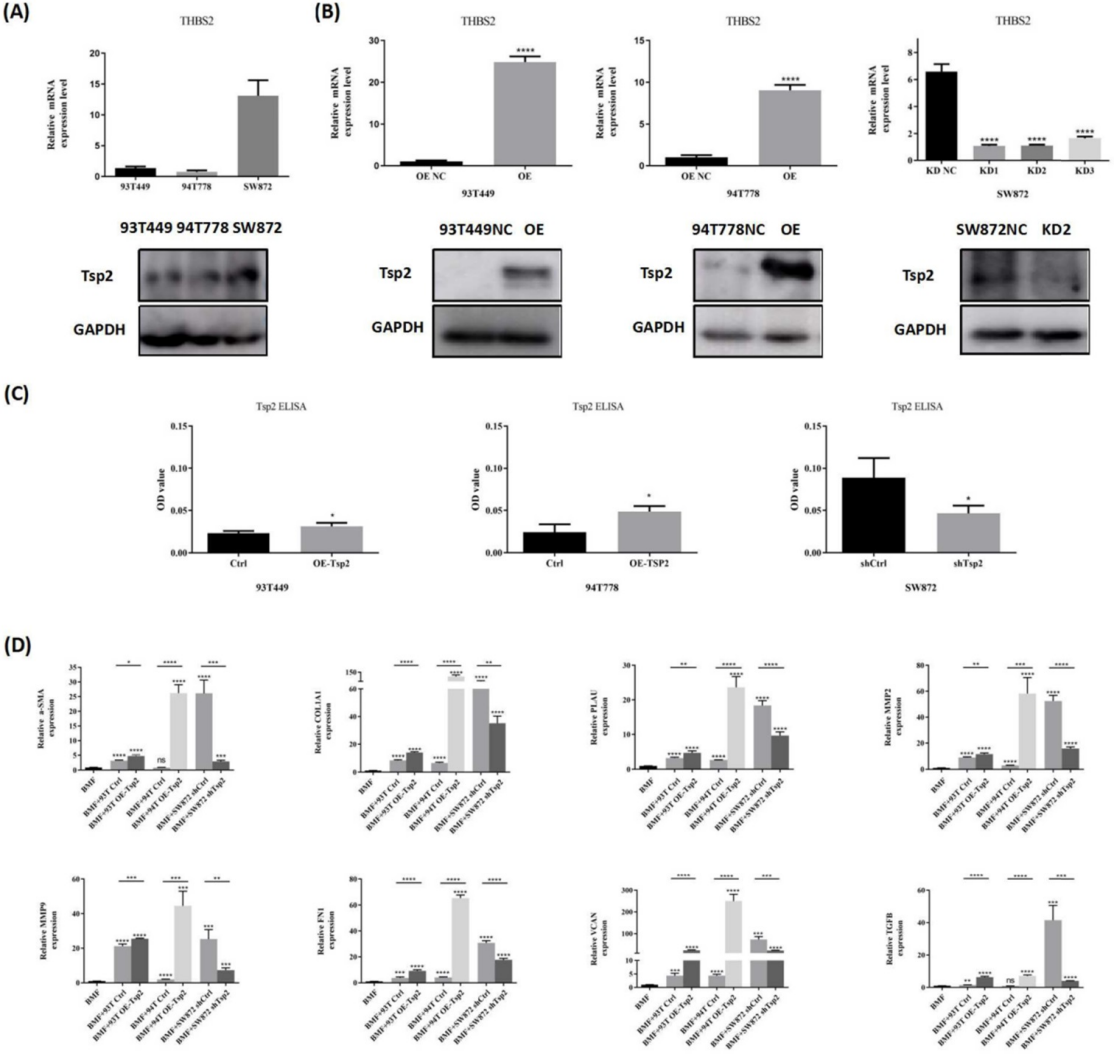
** Tsp2 promoted BMFs transform to TAFs *in vitro.* (A)** Tsp2 expression was lower in 93T449, 94T778 and higher in SW872. **(B)** LPS cells with Tsp2 overexpression or knockdown were constructed and identified (p<0.05). **(C)** Tsp2 protein levels in conditioned medium of LPS cells with Tsp2 overexpression or knockdown were determined by ELISA assay. **(D)** 93T OE-Tsp2 and 94T OE-Tsp2 promoted the transformation of BMFs to TAFs, and SW872 shTsp2 inhibited this transformation (p<0.05). Statistical tests: Student's t tests.

To further investigate whether Tsp2 in RLPS promtes TAFs formation, we constructed LPS cells with stable Tsp2 expression to perform co-culture experiments with BMFs. Specifically, Tsp2 expression was tested by qPCR and western blotting, revealing low Tsp2 expression in 93T449 and 94T778 cells and high Tsp2 expression in SW872 (Fig. 7A). Next, three Tsp2 lentiviral shRNA and control lentivirus were transferred into SW872, and Tsp2 overexpression lentivirus and control vectors were transferred into 93T449 and 94T778. Real-time PCR and western blotting showed that Tsp2 in SW872 was stably knocked down, and Tsp2 in 93T449 and 94T778 was overexpressed (Fig. 7B). SW872 infected with Lenti-shTsp2-2 with the highest knockdown efficiency was used in subsequent experiments.

Tsp2 can be secreted by cells into the matrix for cell-cell interactions. We determined Tsp2 protein levels in CM of LPS cells with Tsp2 overexpression or Tsp2 knockdown by ELISA. The results revealed that compared with control cells, 93T449 and 94T778 with Tsp2 overexpression secreted more Tsp2 to the medium. And SW872 with Tsp2 knockdown secreted less Tsp2 (Fig. 7C). The CM of 93T449 overexpressing Tsp2 (93T OE-Tsp2), 94T778 overexpressing Tsp2 (94T OE-Tsp2), SW872 with Tsp2 knockdown (SW872 shTsp2) and control LPS cells (93T Ctrl, 94T Ctrl and SW872 shCtrl) was collected and co-cultured with BMFs for 24h. The expression levels of TAF markers and secretase in BMFs with or without co-culture with LPS-CM were measured by qPCR. As shown in Fig. 7D, co-culture with CM derived from 93T Ctrl and SW872 shCtrl elevated α-SMA expression in BMFs (p<0.0001). CM of 93T OE-Tsp2 and 94T OE-Tsp2 further promoted α-SMA expression in BMFs (p<0.05, p<0.0001). Compared with SW872 shCtrl, the CM of SW872 shTsp2 reduced the effect of promoting BMFs to express α-SMA (p<0.001). Similarly, after co-culture with CM derived from 93T Ctrl, 94T Ctrl, and SW872 shCtrl, the relative mRNA levels of COL1A1, PLAU, MMP2, MMP9, FN1, VCAN, and TGFB in BMFs enhanced. 93T OE-Tsp2 and 94T OE-Tsp2 promoted the transformation of BMFs into TAFs, and SW872 shTsp2 inhibited this transformation (Fig. 7D). These results indicated that Tsp2 promoted the conversion of TAFs *in vitro* and plays an important role in the formation of TAFs.

### Tsp2 facilitated malignant behaviors of LPS cells

**Figure 8:**
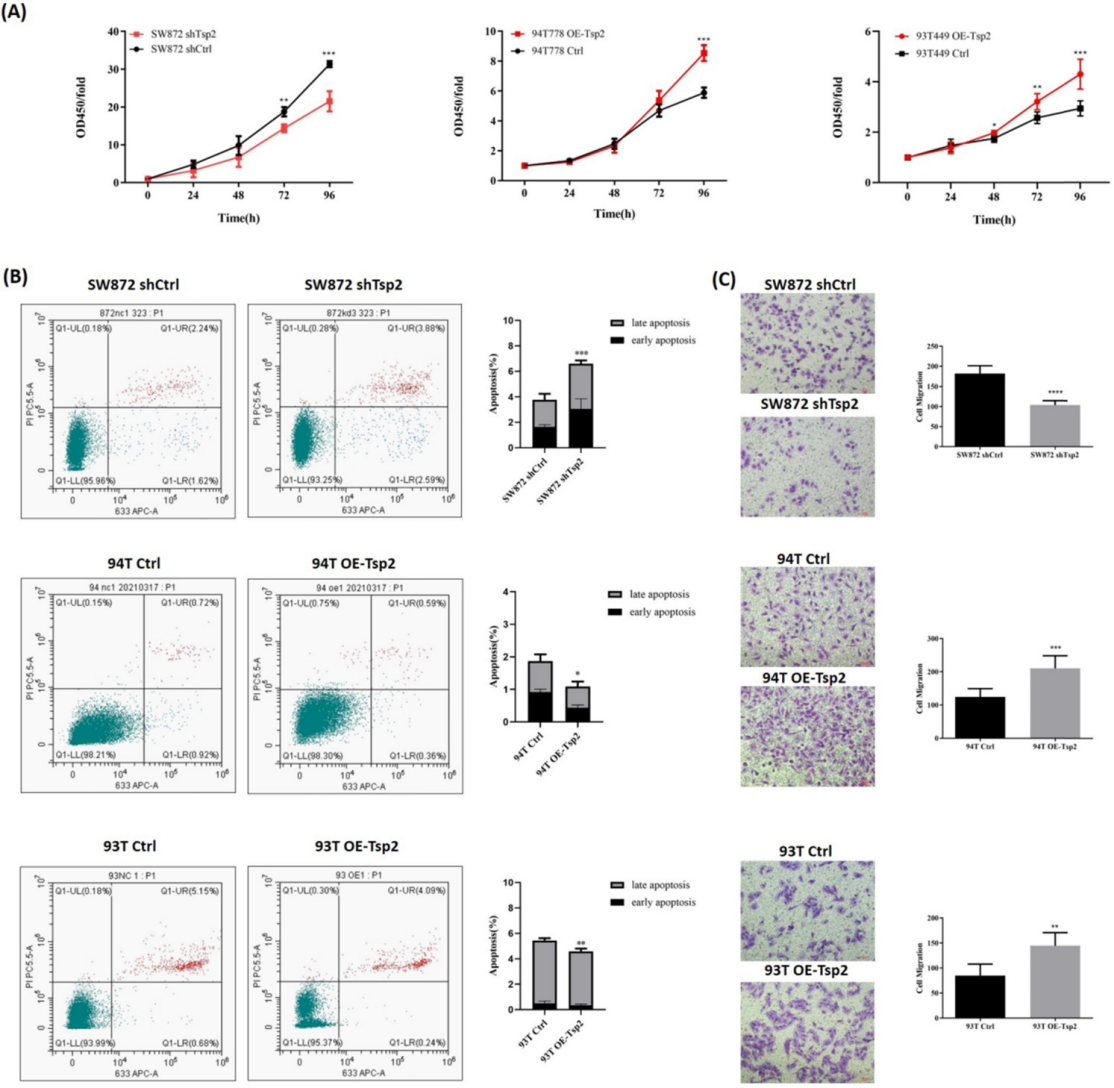
** Tsp2 facilitated malignant behaviors of LPS cells. (A)** Tsp2 downregulation reduced cell proliferation of SW872, and Tsp2 up-regualation enhanced cell proliferation of 93T449 and 94T778 (p<0.05). **(B)** SW872 shTsp2 cells had more apoptotic cells than controls, and LPS cells with Tsp2 overexpression had less apoptotic cells (p<0.05). **(C)** SW872 shTsp2 cells had a lower migration rate, and Tsp2 up-regulation promoted the migration of 93T449 and 94T778 (p<0.05). Statistical tests: Student's t tests.

Cell proliferation, apoptosis, and migration assays were performed to investigate the effect of Tsp2 expression on the biological behaviors of LPS cells. As shown in Fig. 8A, Tsp2 downregulation significantly reduced the proliferation of SW872 cells, while upregulation of Tsp2 enhanced the proliferation of 93T449 and 94T778 cells (p<0.05). Flow cytometry analysis showed that SW872 shTsp2 cells had more apoptotic cells compared with control cells, and LPS cells overexpressing Tsp2 had fewer apoptotic cells (p<0.05, Fig. 8B). The migration assay demonstrated that SW872 shTsp2 had a lower migration rate, and Tsp2 upregulation promoted the migration of 93T449 and 94T778 cells (p<0.05, Fig. 8C).

**Figure 9:**
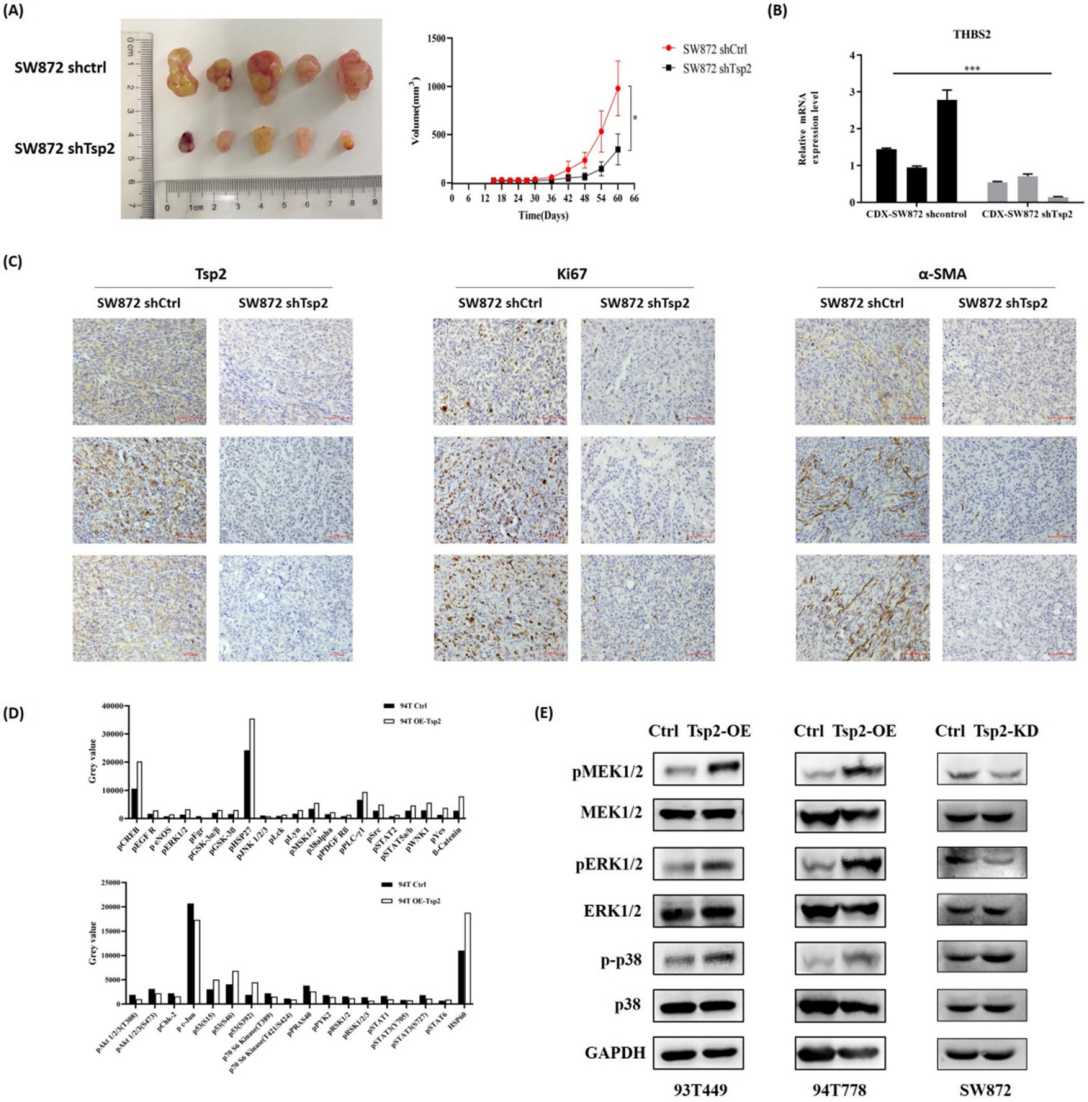
** Tsp2 downregulation suppressed xenograft tumor growth in NOD/SCID mice and the potential mechanism was elucidated by protein microarray. (A)** On day 60, the volume of xenograft tumors formed by SW872 shTsp2 cells were smaller than tumors formed by SW872 shCtrl cells (p<0.05, each group included 5 mice). **(B)** Relative mRNA level of Tsp2 in tumors formed by SW872 shTsp2 cells were decreased (p<0.05) by qPCR. **(C)** Tsp2 and Ki67 expression was weaker, and α-SMA positive cells decreased in SW872 shTsp2 cells derived xenografts. **(D)** The results of protein microarray analysis. **(E)** Western blotting confirmed the variation of pMEK1/2, pERK1/2 and p-p38 expression in Tsp2 overexpression or knockdown cells. Statistical tests: Student's t tests.

Additionally, Tsp2 downregulation suppressed xenograft tumor growth *in vivo* (Fig. 9A). On day 60, the xenograft tumors formed by SW872 shTsp2 cells were smaller than those formed by SW872 shCtrl cells (p<0.05, Fig. 9A). The expression of Tsp2 were lower, Ki67 expression was weaker and α-SMA positive cells decreased in SW872 shTsp2 cell-derived xenografts, suggesting that Tsp2 downregulation inhibited the growth of tumors and TAFs infiltration (Fig. 9B-C).

### Tsp2 activated MAPK/MEK/ERK pathway in LPS cells

A protein microarray was used to evaluate the changes in key targets in tumor-related signaling pathways after Tsp2 overexpression. Compared with control cells, the expression of pCREB, pERK1/2, pMSK1/2, and p-p38alpha were upregulated, and the expression of pAkt1/2/3 and pSTAT1/3 was down-regulated in 94T OE-Tsp2 (Fig. 9D). Western blotting confirmed that Tsp2 upregulation promoted pMEK1/2, pERK1/2, and p-p38 expression in 93T449 and 94T778 cells, while MEK1/2, ERK1/2, and p38 remained unchanged. Knockdown of Tsp2 attenuated pMEK1/2 and pERK1/2 expression in SW872 cells, while the expression of MEK1/2, ERK1/2, p-p38, and p38 was stable (Fig. 9E). Therefore, Tsp2 might activate the MAPK/MEK/ERK pathway to promote the malignant behaviors of LPS cells, and Tsp2 might act as a potential therapeutic target.

## Discussion

Currently, surgical resection remains the most effective treatment for RLPS 23, but postoperative recurrence of RLPS is common, and unresectable local recurrence is associated with tumor-related death 24. The efficacy of radiotherapy and chemotherapy for LPS are controversial 5,6, and targeted therapy has been performed to improve the prognosis of patients. For example, MDM2 inhibitors and CDK4 inhibitors have shown promising prospects, both of which can keep LPS disease stable 25-27; in a phase 2 clinical trial, 44% of 41 RLPS patients (excluding WDLPS) treated with pazopanib showed partial response or stable disease 28. However, Regorafenib and Sunitinib did not show efficacy in LPS 29,30, and the clinical trial results of Aurora kinase A inhibitor alisertib was also unsatisfactory 31. Therefore, it is essential to explore novel therapeutic targets for RLPS and the TME.

The TME is a matrix environment, which consist of stromal cells such as fibroblasts, immune cells, and non-cellular components such as cytokines and extracellular matrix. The supportive effect of TME is essential during cancer progression 32. TAFs are the most important cellular components of the TME and play important roles in tumor development 33. For example, TAFs in gastric cancer and pancreatic cancer secrete CXCL12 and activate the CXCL12/CXCR4-pathway to promote tumor growth 34,35. CCL5 secreted by TAFs can interact with the CCR5 receptor to promote breast cancer metastasis 36. TAFs also secrete collagen, fibronectin, and TGF-β to promote matrix stiffness, release MMPs to modify ECM, and promote the migration and invasion of cancers 37,38. Harati et al. found that TAFs isolated from MLPS and LPS promoted proliferation, viability, and adriamycin resistance of SW872 21, but the role of TAFs and the cross-talk between TAFs and tumor cells are still unclear in RLPS. In our study, we successfully isolated TAFs from retroperitoneal DDLPS and found that they contributed to proliferation and migration of LPS cells and could effectively chemoattract LPS cells.

Surprisingly, unlike the results from colon cancer 7, pancreatic cancer 39 and breast cancer 40, α-SMA expression cannot predict the prognosis of RLPS. Additionally, stable TAFs cannot be isolated from WDLPS, suggesting there might exist special initiating factors of TAFs in DDLPS. Therefore, bioinformatics methods were used to identify the key molecules involved in TAF formation in DDLPS. A total of 193 DEGs were screened, and they were enriched in collagen tissue, smooth muscle cell proliferation, extracellular matrix, and ECM-receptor interaction pathway, demonstrating that there are indeed a group of genes related to TAF formation and ECM proliferation in DDLPS. Using Oncomine database visualization, gene correlation analysis, and qPCR verification, we initially found that THBS2 gene expression was related to TAFs.

Tsp2 protein, which was encoded by THBS2, is a member of the Ca^2+^-binding glycoprotein family of stromal cells, and it interacts with cell receptors and ECM proteins to promote cell adhesion, proliferation, and apoptosis 41. In recent studies, THBS2 has been considered as oncogene or biomarker in tumor development and progression. High Tsp2 expression was significantly related to TNM staging and lymph node metastasis in colon cancer, and it is also a new prognostic indicator of colon cancer 42,43. In lung cancer, gastric cancer, and melanoma, patients with positive Tsp2 expression have poorer prognosis 44-46. However, it is not fully understood whether the effect of Tsp2 in promoting tumor progression and ECM hyperplasia is related to the activation of TAFs. It is only found in breast cancer that tumor-initiating cells activate fibroblasts by secreting Tsp2 to enhance metastasis 47. Bornstein et al. knocked-out Tsp2 in mice, and then observed apoptosis of fibroblasts and abnormal structure of collagen fiber 48. Here, consistent with the results of other tumor types, Tsp2 expression is an independent predictive factor of DFS and RFS in RLPS. Tsp2 expression was also positively correlated with α-SMA expression in RLPS patients. In addition, Tsp2 promoted the conversion of BMFs to TAFs *in vitro* and enhanced the expression of α-SMA, TAFs secretase, and ECM components. Reduction of Tsp2 in the co-culture environment inhibited TAF formation induced by LPS cell lines. α-SMA positive cells also decreased in SW872 shTsp2 cell-derived xenografts. Therefore, Tsp2 might participate in transformation to TAFs and be an effective marker for TAFs infiltration and prognosis prediction in RLPS.

The bidirectional effect between TAFs and tumor cells plays a key role in the development and progression of LPS. Firstly, tumor cells secrete Tsp2 to induce the transformation of normal fibroblasts to TAFs, and TAFs remodel the extracellular matrix by secreting COL1A1, COL1A2, COL5A1, FN1, and VCAN. Proliferating stroma cells and deposited collagen may form a tough barrier around the tumor cells to prevent immune cell infiltration and drug arrival 49,50. MMPs and other proteases secreted by TAFs dissolve the proliferating extracellular matrix and provide channels for tumor invasion, implantation and metastasis 9,51. Secondly, TAFs could regulate the malignant biological behavior of tumor cells directly 52,53, which consistent with the results observed in this study. Thirdly, RLPS tumor tissue is difficult to completely resected and prone to recurrence repeatedly 3, TAFs in the resection margin or adjacent tissues may promote the proliferation of residual tumor cells and thus lead to tumor recurrence. Patricia P et al found that detecting THBS2 and other gene expression at negative surgical margins could predict the recurrence of oral squamous cell carcinoma 54. In this study, we confirmed that RLPS patients with high Tsp2 expression had shorter DFS and RFS, and the expression of TAFs-related genes and THBS2 at the resection margin of RLPS should be further evaluated to support this hypothesis. Thus, the crosstalk between TAFs and LPS cells forms a positive feedback. At present, it is difficult to determine the sequence of tumorigenesis and TAFs formation, but once the positive feedback is initiated, it will lead to malignant changes of tumor cells and the tumor microenvironment as a whole.

Here, the function of Tsp2 in LPS cells was investigated. Similar to previous studies in gastric cancer cells and melanoma cells 46,55, knockdown of Tsp2 suppressed the malignant biological behaviors of LPS cells and reduced the volume of SW872 derived xenograft tumors. Unlike the discovery that Tsp2 deletion inhibited the AKT/PI3K pathway in gastric cancer and melanoma, we found that pCREB, pERK1/2, pMSK1/2, and p-p38alpha were up-regulated, pAkt1/2/3 and pSTAT1/3 were down-regulated in 94T OE-Tsp2 through the protein microarray analysis. MSK and CREB are downstream kinases of the MEK/ERK pathway, which mediate downstream gene transcription and participate in regulating biological events. JNK and p38 signaling pathways are important in stress responses, such as inflammation and apoptosis. Western blotting confirmed that Tsp2 upregulation promoted pMEK1/2, pERK1/2, and p-p38 expression, while Tsp2 knockdown attenuated pMEK1/2 and pERK1/2 expression. Therefore, we defer that Tsp2 can activate the MAPK/MEK/ERK pathway to facilitate tumor progression. Currently, single or combined therapies of MAPK inhibitors Trametinib, Abrafenib, and Vemurafenib have been used in BRAF V600-mutated melanoma 56-58, BRAF-mutated non-small cell lung cancer 59,60 and anaplastic thyroid cancer 61. Other drugs targeting MAPK such as ERK1/2 inhibitor ulixertinib have also shown potent activity in clinical trials including colon cancer, gallbladder adenocarcinoma, glioblastoma and other solid tumors 62. Therefore, MAPK inhibitors might be an effective therapy for RLPS, and the specific application methods and possible effect need to be determined by clinical trials.

Since Tsp2 is a type of secreted protein, assessment of Tsp2 in serum might also indicate the grade and prognosis of tumor, or increase the detection rate of patients with high-risk tumors 63,64. However, limited to the number of blood samples, we did not evaluate Tsp2 expression in the serum of patients, although this may be a more convenient and effective inspection method. Additionally, the specific mechanism by which Tsp2 promotes TAF formation remains to be elucidated, and we will elucidate these problems in future studies.

In summary, we isolated and identified TAFs from retroperitoneal DDLPS and determined its cancer-promoting effect. Tsp2 promoted TAF formation and tumor progression and was an independent predictive factor for DFS and RFS in RLPS. Therefore, Tsp2 may be a promising therapeutic target for RLPS patients.

## Supplementary Material

Supplementary tables.

## Figures and Tables

**Table 1:** Correlation between α-SMA expression and clinicopathological features in 112 RLPS patients

Clinicopathological features	a-SMA Positive Expression (n, %)	a-SMA Negative Expression (n, %)	p-value
**Gender**			0.697
Male	41 (65.1)	22 (34.9)	
Female	30 (61.2)	19 (38.8)	
**Age**			0.843
<60	41 (62.1)	25 (37.9)	
≥60	30 (65.2)	16 (34.8)	
**Diameter**			0.002**
≤22	45 (77.6)	13 (22.4)	
>22	26 (48.2)	28 (51.8)	
**Grade**			0.004**
Low (G1)	9 (37.5)	15 (62.5)	
High (G2, G3)	62 (70.5)	26 (29.5)	
**Tumor subtype**			0.005**
WDLPS	9 (37.5)	15 (62.5)	
DDLPS	54 (72.0)	21 (28.0)	
PLS	7 (77.8)	2 (22.2)	
MLPS	1 (25.0)	3 (75.0)	
**Vascular invasion^a^**			0.551
No	67 (63.2)	39 (36.8)	
Yes	3 (100.0)	0	
**Lymph node metastasis^a^**			0.531
No	67 (62.6)	40 (37.4)	
Yes	2 (100.0)	0	
**Organs invasion^a^**			0.280
No	17 (54.8)	14 (45.2)	
Yes	51 (66.2)	26 (33.8)	
**Primary/recurrence**			0.846
Primary	33 (62.3)	20 (37.7)	
Recurrence	38 (64.4)	21 (35.6)	

**P<0.01; ^a^ Patients without relevant information were not included in statistics.

**Table 2:** Cox proportional hazards regression model analysis of DFS in RLPS patients

Clinicopathological features	Univariate Analysis		Multivariate Analysis	
	HR (95%CI)	p-value	HR (95%CI)	p-value
**Tsp2**				
Negative	1		1	
Positive	1.815 (1.061-3.106)	0.030*	1.715 (1.001-2.941)	0.0497*
**a-SMA**				
Negative	1			
Positive	1.281 (0.760-2.160)	0.352		
**Gender**				
Male	1			
Female	1.113 (0.673-1.842)	0.676		
**Age(years)**	1.013 (0.992-1.035)	0.222		
**Diameter (cm)**	1.013 (0.997-1.028)	0.106		
**Grade**				
G1	1			
G2	2.375 (1.073-5.255)	0.033*		
G3	2.667 (1.218-5.838)	0.014*		
**Tumor type**				
WDLPS	1			
DDLPS	2.294 (1.083-4.861)	0.030*		
PLS	1.845 (0.668-5.094)	0.237		
MLPS	6.704 (1.389-32.351)	0.018*		
**Vascular invasion**				
No	1			
Yes	1.343 (0.326-5.524)	0.683		
**Lymph node metastasis**				
No	1			
Yes	3.052 (0.736-12.663)	0.124		
**Organs invasion**				
No	1		1	
Yes	2.385 (1.313-4.331)	0.004**	2.248 (1.235-4.092)	0.008**
**Primary/recurrence**				
Primary	1			
Recurrence	1.396 (0.855-2.279)	0.182		

*P<0.05; **P<0.01.

**Table 3:** Cox proportional hazards regression model analysis of RFS in RLPS patients

Clinicopathological features	Univariate Analysis		Multivariate Analysis	
	HR (95%CI)	p-value	HR (95%CI)	p-value
**Tsp2**				
Negative	1		1	
Positive	2.107 (1.115-3.983)	0.022*	2.000 (1.056-3.786)	0.033*
**a-SMA**				
Negative	1			
Positive	1.545 (0.829-2.879)	0.170		
**Gender**				
Male	1			
Female	1.026 (0.572-1.840)	0.931		
Age (years)	1.013 (0.989-1.038)	0.298		
Diameter (cm)	1.002 (0.983-1.022)	0.811		
**Grade**				
G1	1			
G2	2.156 (0.854-5.441)	0.104		
G3	2.856 (1.165-7.002)	0.022*		
**Tumor type**				
WDLPS	1			
DDLPS	1.855 (0.821-4.195)	0.138		
PLS	1.733 (0.582-5.160)	0.323		
MLPS	8.807 (1.764-43.966)	0.008**		
**Vascular invasion**				
No	1			
Yes	0.875 (0.120-6.379)	0.895		
**Lymph node metastasis**				
No	1		1	
Yes	4.518 (1.072-19.045)	0.040*	2.537 (1.256-5.125)	0.009**
**Organs invasion**				
No	1			
Yes	2.694 (1.337-5.428)	0.006**		
**Primary/recurrence**				
Primary	1			
Recurrence	1.343 (0.763-2.362)	0.306		

*P<0.05; **P<0.01.

## References

[1] Gronchi A, Miceli R, Shurell E (2013). Outcome prediction in primary resected retroperitoneal soft tissue sarcoma: histology-specific overall survival and disease-free survival nomograms built on major sarcoma center data sets. Journal of clinical oncology: official journal of the American Society of Clinical Oncology.

[2] Sbaraglia M, Bellan E, Dei Tos AP (2021). The 2020 WHO Classification of Soft Tissue Tumours: news and perspectives. Pathologica.

[3] Crago AM, Singer S (2011). Clinical and molecular approaches to well differentiated and dedifferentiated liposarcoma. Current opinion in oncology.

[4] Lewis JJ, Leung D, Woodruff JM (1998). Retroperitoneal soft-tissue sarcoma: analysis of 500 patients treated and followed at a single institution. Annals of surgery.

[5] Bramwell VH, Anderson D, Charette ML (2003). Doxorubicin-based chemotherapy for the palliative treatment of adult patients with locally advanced or metastatic soft tissue sarcoma. The Cochrane database of systematic reviews.

[6] Baldini EH, Wang D, Haas RL (2015). Treatment Guidelines for Preoperative Radiation Therapy for Retroperitoneal Sarcoma: Preliminary Consensus of an International Expert Panel. International journal of radiation oncology, biology, physics.

[7] Sha M, Jeong S, Qiu BJ (2018). Isolation of cancer-associated fibroblasts and its promotion to the progression of intrahepatic cholangiocarcinoma. Cancer medicine.

[8] Orimo A, Gupta PB, Sgroi DC (2005). Stromal fibroblasts present in invasive human breast carcinomas promote tumor growth and angiogenesis through elevated SDF-1/CXCL12 secretion. Cell.

[9] Kalluri R, Zeisberg M (2006). Fibroblasts in cancer. Nature reviews. Cancer.

[10] Valencia T, Kim JY, Abu-Baker S (2014). Metabolic reprogramming of stromal fibroblasts through p62-mTORC1 signaling promotes inflammation and tumorigenesis. Cancer cell.

[11] LeBleu VS, Kalluri R (2018). A peek into cancer-associated fibroblasts: origins, functions and translational impact. Disease models & mechanisms.

[12] Lakins MA, Ghorani E, Munir H (2018). Cancer-associated fibroblasts induce antigen-specific deletion of CD8 (+) T Cells to protect tumour cells. Nature communications.

[13] Erdogan B, Webb DJ (2017). Cancer-associated fibroblasts modulate growth factor signaling and extracellular matrix remodeling to regulate tumor metastasis. Biochemical Society transactions.

[14] Mohan V, Das A, Sagi I (2020). Emerging roles of ECM remodeling processes in cancer. Seminars in cancer biology.

[15] Albrengues J, Bertero T, Grasset E (2015). Epigenetic switch drives the conversion of fibroblasts into proinvasive cancer-associated fibroblasts. Nature communications.

[16] Ringuette Goulet C, Bernard G, Tremblay S (2018). Exosomes Induce Fibroblast Differentiation into Cancer-Associated Fibroblasts through TGFβ Signaling. Molecular cancer research: MCR.

[17] Lim EJ, Suh Y, Kim S (2018). Force-mediated proinvasive matrix remodeling driven by tumor-associated mesenchymal stem-like cells in glioblastoma. BMB reports.

[18] Luo H, Tu G, Liu Z (2015). Cancer-associated fibroblasts: a multifaceted driver of breast cancer progression. Cancer letters.

[19] Öhlund D, Elyada E, Tuveson D (2014). Fibroblast heterogeneity in the cancer wound. The Journal of experimental medicine.

[20] Quail DF, Joyce JA (2013). Microenvironmental regulation of tumor progression and metastasis. Nature medicine.

[21] Harati K, Daigeler A, Hirsch T (2016). Tumor-associated fibroblasts promote the proliferation and decrease the doxorubicin sensitivity of liposarcoma cells. International journal of molecular medicine.

[22] Jung Y, Kim JK, Shiozawa Y (2013). Recruitment of mesenchymal stem cells into prostate tumours promotes metastasis. Nature communications.

[23] Dumitra S, Gronchi A (2018). The Diagnosis and Management of Retroperitoneal Sarcoma. Oncology (Williston Park, N.Y.).

[24] Bagaria SP, Gabriel E, Mann GN (2018). Multiply recurrent retroperitoneal liposarcoma. Journal of surgical oncology.

[25] Ray-Coquard I, Blay JY, Italiano A (2012). Effect of the MDM2 antagonist RG7112 on the P53 pathway in patients with MDM2-amplified, well-differentiated or dedifferentiated liposarcoma: an exploratory proof-of-mechanism study. The Lancet. Oncology.

[26] de Jonge M, de Weger VA, Dickson MA (2017). A phase I study of SAR405838, a novel human double minute 2 (HDM2) antagonist, in patients with solid tumours. European journal of cancer (Oxford, England: 1990).

[27] Dickson MA, Schwartz GK, Keohan ML (2016). Progression-Free Survival Among Patients With Well-Differentiated or Dedifferentiated Liposarcoma Treated With CDK4 Inhibitor Palbociclib: A Phase 2 Clinical Trial. JAMA oncology.

[28] Samuels BL, Chawla SP, Somaiah N (2017). Results of a prospective phase 2 study of pazopanib in patients with advanced intermediate-grade or high-grade liposarcoma. Cancer.

[29] Mir O, Brodowicz T, Italiano A (2016). Safety and efficacy of regorafenib in patients with advanced soft tissue sarcoma (REGOSARC): a randomised, double-blind, placebo-controlled, phase 2 trial. The Lancet. Oncology.

[30] Mahmood ST, Agresta S, Vigil CE (2011). Phase II study of sunitinib malate, a multitargeted tyrosine kinase inhibitor in patients with relapsed or refractory soft tissue sarcomas. Focus on three prevalent histologies: leiomyosarcoma, liposarcoma and malignant fibrous histiocytoma. International journal of cancer.

[31] Dickson MA, Mahoney MR, Tap WD (2016). Phase II study of MLN8237 (Alisertib) in advanced/metastatic sarcoma. Annals of oncology: official journal of the European Society for Medical Oncology.

[32] Lorusso G, Rüegg C (2008). The tumor microenvironment and its contribution to tumor evolution toward metastasis. Histochemistry and cell biology.

[33] Erez N, Truitt M, Olson P (2010). Cancer-Associated Fibroblasts Are Activated in Incipient Neoplasia to Orchestrate Tumor-Promoting Inflammation in an NF-kappaB-Dependent Manner. Cancer cell.

[34] Izumi D, Ishimoto T, Miyake K (2016). CXCL12/CXCR4 activation by cancer-associated fibroblasts promotes integrin β1 clustering and invasiveness in gastric cancer. International journal of cancer.

[35] Li X, Ma Q, Xu Q (2012). SDF-1/CXCR4 signaling induces pancreatic cancer cell invasion and epithelial-mesenchymal transition *in vitro* through non-canonical activation of Hedgehog pathway. Cancer letters.

[36] Karnoub AE, Dash AB, Vo AP (2007). Mesenchymal stem cells within tumour stroma promote breast cancer metastasis. Nature.

[37] Lee HO, Mullins SR, Franco-Barraza J (2011). FAP-overexpressing fibroblasts produce an extracellular matrix that enhances invasive velocity and directionality of pancreatic cancer cells. BMC cancer.

[38] Paauwe M, Schoonderwoerd MJA, Helderman R (2018). Endoglin Expression on Cancer-Associated Fibroblasts Regulates Invasion and Stimulates Colorectal Cancer Metastasis. Clinical cancer research: an official journal of the American Association for Cancer Research.

[39] Fujita H, Ohuchida K, Mizumoto K (2010). alpha-Smooth Muscle Actin Expressing Stroma Promotes an Aggressive Tumor Biology in Pancreatic Ductal Adenocarcinoma. Pancreas.

[40] Yamashita M, Ogawa T, Zhang X (2012). Role of stromal myofibroblasts in invasive breast cancer: stromal expression of alpha-smooth muscle actin correlates with worse clinical outcome. Breast cancer (Tokyo, Japan).

[41] Bornstein P, Armstrong LC, Hankenson KD (2000). Thrombospondin 2, a matricellular protein with diverse functions. Matrix biology: journal of the International Society for Matrix Biology.

[42] Tian Q, Liu Y, Zhang Y (2018). THBS2 is a biomarker for AJCC stages and a strong prognostic indicator in colorectal cancer. Journal of B.U.ON.: official journal of the Balkan Union of Oncology.

[43] Wang X, Zhang L, Li H (2016). THBS2 is a Potential Prognostic Biomarker in Colorectal Cancer. Scientific reports.

[44] Weng TY, Wang CY, Hung YH (2016). Differential Expression Pattern of THBS1 and THBS2 in Lung Cancer: Clinical Outcome and a Systematic-Analysis of Microarray Databases. PloS one.

[45] Zhuo C, Li X, Zhuang H (2016). Elevated THBS2, COL1A2, and SPP1 Expression Levels as Predictors of Gastric Cancer Prognosis. Cellular physiology and biochemistry: international journal of experimental cellular physiology, biochemistry, and pharmacology.

[46] Liu QH, Ma LS (2018). Knockdown of thrombospondin 2 inhibits metastasis through modulation of PI3K signaling pathway in uveal melanoma cell line M23. European review for medical and pharmacological sciences.

[47] Del Pozo Martin Y, Park D, Ramachandran A (2015). Mesenchymal Cancer Cell-Stroma Crosstalk Promotes Niche Activation, Epithelial Reversion, and Metastatic Colonization. Cell reports.

[48] Bornstein P, Kyriakides TR, Yang Z (2000). Thrombospondin 2 modulates collagen fibrillogenesis and angiogenesis. The journal of investigative dermatology. Symposium proceedings.

[49] Chauhan VP, Stylianopoulos T, Boucher Y (2011). Delivery of molecular and nanoscale medicine to tumors: transport barriers and strategies. Annual review of chemical and biomolecular engineering.

[50] Correia AL, Bissell MJ (2012). The tumor microenvironment is a dominant force in multidrug resistance. Drug resistance updates: reviews and commentaries in antimicrobial and anticancer chemotherapy.

[51] Kim H, Watkinson J, Varadan V (2010). Multi-cancer computational analysis reveals invasion-associated variant of desmoplastic reaction involving INHBA, THBS2 and COL11A1. BMC medical genomics.

[52] Luga V, Zhang L, Viloria-Petit AM (2012). Exosomes mediate stromal mobilization of autocrine Wnt-PCP signaling in breast cancer cell migration. Cell.

[53] Bissell MJ, Hines WC (2011). Why don't we get more cancer? A proposed role of the microenvironment in restraining cancer progression. Nature medicine.

[54] Reis PP, Tokar T, Goswami RS (2020). A 4-gene signature from histologically normal surgical margins predicts local recurrence in patients with oral carcinoma: clinical validation. Scientific reports.

[55] Ao R, Guan L, Wang Y (2018). Silencing of COL1A2, COL6A3, and THBS2 inhibits gastric cancer cell proliferation, migration, and invasion while promoting apoptosis through the PI3k-Akt signaling pathway. Journal of cellular biochemistry.

[56] Flaherty KT, Robert C, Hersey P (2012). Improved survival with MEK inhibition in BRAF-mutated melanoma. The New England journal of medicine.

[57] Hauschild A, Grob JJ, Demidov LV (2012). Dabrafenib in BRAF-mutated metastatic melanoma: a multicentre, open-label, phase 3 randomised controlled trial. Lancet (London, England).

[58] Sosman JA, Kim KB, Schuchter L (2012). Survival in BRAF V600-mutant advanced melanoma treated with vemurafenib. The New England journal of medicine.

[59] Kotani H, Adachi Y, Kitai H (2018). Distinct dependencies on receptor tyrosine kinases in the regulation of MAPK signaling between BRAF V600E and non-V600E mutant lung cancers. Oncogene.

[60] Planchard D, Besse B, Groen HJM (2016). Dabrafenib plus trametinib in patients with previously treated BRAF(V600E)-mutant metastatic non-small cell lung cancer: an open-label, multicentre phase 2 trial. The Lancet. Oncology.

[61] Cabanillas ME, Ryder M, Jimenez C (2019). Targeted Therapy for Advanced Thyroid Cancer: Kinase Inhibitors and Beyond. Endocrine reviews.

[62] Sullivan RJ, Infante JR, Janku F (2018). First-in-Class ERK1/2 Inhibitor Ulixertinib (BVD-523) in Patients with MAPK Mutant Advanced Solid Tumors: Results of a Phase I Dose-Escalation and Expansion Study. Cancer discovery.

[63] Le Large TYS, Meijer LL, Paleckyte R (2020). Combined Expression of Plasma Thrombospondin-2 and CA19-9 for Diagnosis of Pancreatic Cancer and Distal Cholangiocarcinoma: A Proteome Approach. The oncologist.

[64] Kim J, Bamlet WR, Oberg AL (2017). Detection of early pancreatic ductal adenocarcinoma with thrombospondin-2 and CA19-9 blood markers. Science translational medicine.

