# Supplementary material for: Tsp2 Facilitates Tumor-associated Fibroblasts Formation and Promotes Tumor Progression in Retroperitoneal Liposarcoma
Source: Int J Biol Sci. 2022 Aug 1;18(13):5038–55. doi: 10.7150/ijbs.70083 (PMC9379409; doi:10.7150/ijbs.70083)
Supplement: Supplementary file 1 — Supplementary tables. [file ijbsv18p5038s1.pdf]

Supplementary Tables

Table S1. Clinicopathological features of 112 RLPS patients

Clinicopathological features	Cases
**Gender**	
Male	63
Female	49
**Age**	
<60	66
⩾60	46
**Diameter**	
⩽22	58
>22	54
**Grade**	
Low（G1）	24
High (G2、G3）	88
**Tumor type**	
WDLPS	24
DDLPS	75
PLS	9
MLPS	4
**Vascular invasion**[a]	
No	106
Yes	3
**Lymph node metastasis**[a]	
No	107
Yes	2
**Organs invasion**[a]	

No	31
Yes	77
**Primary/recurrence**	
Primary	53
Recurrence	59

[a] Patients without relevant information were not included in statistics.

Table S2. Primers used for PCR amplification

Gene Symbol	Forward (5'-3')	Reverse (3'-5')
FAP	CAAAGGCTGGAGCTAAGAATCC	ACTGCAAACATACTCGTTCATCA
ACTA2	AAAAGACAGCTACGTGGGTGA	GCCATGTTCTATCGGGTACTTC
THBS2	GACACGCTGGATCTCACCTAC	GAAGCTGTCTATGAGGTCGCA
COL1A1	GAGGGCCAAGACGAAGACATC	CAGATCACGTCATCGCACAAC
COL1A2	GAGCGGTAACAAGGGTGAGC	CTTCCCCATTAGGGCCTCTC
COL5A1	TACAACGAGCAGGGTATCCAG	ACTTGCCATCTGACAGGTTGA
FN1	AGGAAGCCGAGGTTTTAACTG	AGGACGCTCATAAGTGTCACC
VCAN	GTAACCCATGCGCTACATAAAGT	GGCAAAGTAGGCATCGTTGAAA
PLAU	CTGTCACCTACGTGTGTGGAG	TGAGCGACCCAGGTAGACG
MMP2	CCCACTGCGGTTTTCTCGAAT	CAAAGGGGTATCCATCGCCAT
MMP9	AGACCTGGGCAGATTCCAAAC	CGGCAAGTCTTCCGAGTAGT
MMP11	AAGGTATGGAGCGATGTGACG	GTCCAGGTCTCATCATAGTCGAA
TGFB	CTAATGGTGGAAACCCACAACG	TATCGCCAGGAATTGTTGCTG
GAPDH	AACTTTGGTATCGTGGAAGGAC	CAGTAGAGGCAGGGATGATGTT

Table S3. Antibodies for western blotting

Antibody	Company	Catalog No.	Dilution
Tsp2	Invitrogen, USA	PA5-80123	0.1μg/ml
GAPDH	Abmart, China	M20006	1:5000

pMEK1/2(Ser217/221)	CST, USA	#9154	1:1000
MEK1/2	CST, USA	#8727	1:1000
pERK1/2(Thr202/Tyr204)	Abcam, USA	ab214362	1:1000
ERK1/2	Abcam, USA	ab196883	1:1000
p-p38(Thr180/Tyr182)	CST, USA	#9211	1:1000
p38	CST, USA	#8690	1:1000

Table S4. Clinicopathological characteristics of RLPS patients from whom TAFs were isolated

**Clinicopathological characteristics**	**Patient 1**	**Patient 2**	**Patient 3**	**Patient 4**
Tumor type	DDLPS	WDLPS	DDLPS	WDLPS
Gender	Male	Female	Male	Female
Age (years)	51	57	63	63
Grade	3	1	3	1
Vascular invasion	No	No	No	No
Lymph node metastasis	No	No	No	No
Organs invasion	Yes	Yes	No	Yes
Primary/recurrence	Primary	Recurrent	Recurrent	Recurrent
Results of isolate TAFs	TAF1	Failed	TAF2	Failed

Table S5. Correlation between Tsp2 expression and clinicopathological features in 112 RLPS patients

**Clinicopathological features**	**Tsp2 Positive Expression (n,%)**	**Tsp2 Negtive Expression (n,%)**	**P-value**
**Gender**			0.698
Male	40(63.5)	23(36.5)	
Female	29(59.2)	20(40.8)	
**Age**			**0.031***
<60	35(53.0)	31(47.0)	
⩾60	34(73.9)	12(26.1)	

**Diameter**			0.052
≤22	41(70.7)	17(29.3)	
>22	28(51.9)	26(48.1)	
**Grade**			
Low (G1)	11(45.8)	13(54.2)	0.073
High (G2、G3)	58(65.9)	30(34.1)	
**Tumor subtype**			0.123
WDLPS	10(41.7)	14(58.3)	
DDLPS	49(65.3)	26(34.7)	
PLS	7(77.8)	2(22.2)	
MLPS	3(75.0)	1(25.0)	
**Vascular invasion**[a]			>0.9999
No	64(60.4)	42(39.6)	
Yes	2(66.7)	1(33.3)	
**Lymph node metastasis**[a]			0.519
No	64(59.8)	43(40.2)	
Yes	2(100.0)	0	
**Organs invasion**[a]			0.275
No	16(51.6)	15(48.4)	
Yes	50(64.9)	27(35.1)	
**Primary/recurrence**			0.437
Primary	35(66.0)	18(34.0)	
Recurrence	34(57.6)	25(42.4)	

[a] Patients without relevant information were not included in statistics.
